# Autologous cell lines from circulating colon cancer cells captured from sequential liquid biopsies as model to study therapy-driven tumor changes

**DOI:** 10.1038/s41598-018-34365-z

**Published:** 2018-10-29

**Authors:** Alexandra Soler, Laure Cayrefourcq, Thibault Mazard, Anna Babayan, Pierre-Jean Lamy, Said Assou, Eric Assenat, Klaus Pantel, Catherine Alix-Panabières

**Affiliations:** 1 Laboratory of Rare Human Circulating Cells, University Medical Centre of Montpellier, EA2415, Montpellier, France; 2 Department of Medical Oncology, Cancer Institute of Montpellier, Montpellier, France; 3 Department of Tumor Biology, University Medical Centre Hamburg-Eppendorf, Hamburg, Germany; 4 Imagenome, Labosud, Montpellier, France; 5 IRMB, Montpellier University, INSERM U1183, University Medical Centre of Montpellier, Montpellier, France; 6 Department of Medical Oncology, University Medical Centre of Montpellier, Montpellier, France

## Abstract

Circulating tumor cells (CTCs) are important clinical indicators for prognosis and treatment efficacy. However, CTC investigation is hampered by their low number, making the establishment of permanent CTC lines very challenging. We derived and characterized nine CTC lines using blood samples from a patient with metastatic colorectal cancer collected before and after chemotherapy and targeted therapy, and during cancer progression. These cell lines displayed an intermediate epithelial/mesenchymal phenotype, stem-cell like characteristics, angiogenesis potential, an osteomimetic signature and the capacity to escape from the immune system. Moreover, they showed changes in mRNA and protein expression (e.g., *DEFA6*, *ABCB1* and *GAL*), whereas analysis of chromosomal copy number aberrations revealed no significant variation over time. These data indicate that although CTC lines derived from sequential blood samples during therapy have common traits, treatment-resistant CTC clones with distinct phenotypic characteristics are selected over time.

## Introduction

Over the past years, liquid biopsy of circulating tumor cells (CTC) has received major attention as a strategy to obtain real time diagnostic and prognostic information^1,2^. Several large-scale clinical trials and meta-analyses have shown that CTC number is an important indicator of the risk of cancer progression or death in patients with metastatic solid tumors^3–7^. However, CTC investigation is hampered by their very low number, especially in blood of patients with colorectal cancer. Therefore, the establishment of *in vitro* cultures and permanent lines from CTCs has become the most challenging task over the past years. Indeed, CTC lines could be used to identify proteins and pathways involved in cancer cell stemness and dissemination, and also to test new drugs to inhibit metastasis-competent CTCs. *Ex vivo* CTC cultures have been established for breast^8,9^, prostate^10^, lung^11^, and head and neck cancer^12^. We previously described the first permanent cell line (CTC-MCC-41) from circulating colon cancer cells^13^. However, its establishment was very difficult (blood samples of 168 patients were tested). This could be partly explained by the much lower CTC number in the peripheral blood of such patients than in patients with breast or prostate cancer, making very difficult their enrichment and culture. In addition to its capacity to expand *in vitro* for more than 4 years, the CTC-MCC-41 cell line shows specific stem-cell like characteristics and shares some features of the original primary tumor and lymph node metastasis^13^.

We then established another eight CTC lines from blood samples collected from the same patient at different time points during his follow-up. This unique biological material represents a chance to study clonal selection and resistance mechanisms during tumor progression and treatment. Here, we report the establishment of these new CTC lines from the same patient with metastatic colon cancer, and their characterization (genome, transcriptome, proteome, and functional analyses). Comparison of all nine autologous CTC lines (the previously described CTC-MCC-41 line and the eight new lines) highlighted their common features and main differences acquired over time.

## Results

### Establishment of colon CTC lines from a patient with metastatic colon cancer

**Figure 1:**
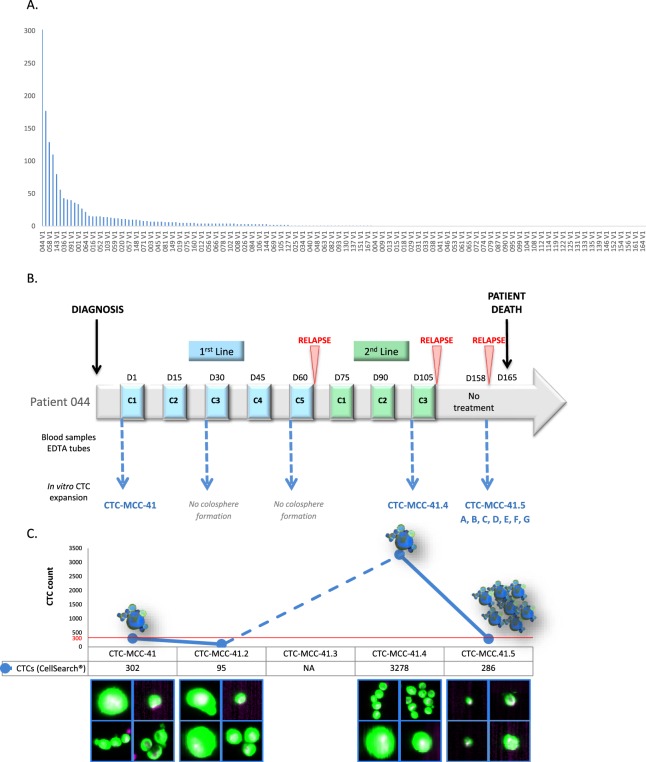
Blood samples collection for the establishment of CTC-derived colospheres and timeline of CTC line derivation from sequential blood samples of patient 044. (**A**) CTC number (assessed with the CellSearch system) in the blood sample of the 168 patients with metastatic colon cancer at V1 (before any treatment); (**B**) Nine colon CTC lines were established from blood samples of patient 044 at different time points: before treatment initiation (CTC-MCC-41 line), at the time of the second relapse at the end of second-line therapy (CTC-MCC-41.4 line), and at the time of the third relapse before the patient death (CTC-MCC-41.5 A-G lines); D, day; C, treatment cycle; (**C**) Correlation between CTC number detected with the CellSearch system and *in vitro* colosphere formation (in blue) for patient 044; CellSearch cell images representative of CTC morphology are shown for the blood samples from which CTC lines could be derived (cells in green). The red line shows the cut-off of approximately 300 cells per 7.5 mL of blood required for *in vitro* CTC expansion.

The national COLOSPOT project included 168 patients with metastatic colon cancer (NCT01596790). Before the first-line treatment, CTC number was evaluated in 7.5 mL of peripheral blood using the CellSearch system, and then another 10 mL of peripheral blood was used for CTC enrichment and culture. CTC number was ≥1 in 57.5% of patients and ≥3 in 39.4% (mean = 9; median = 1; range: 0-302). Only one colon CTC line (CTC-MCC-41) could be established^13^ from the patient with the highest CTC number (302 CTCs/7.5 mL of blood) and with 38 CTC clusters (<2 to 5 CTCs/microemboli) (patient 044) (Fig. 1A).

During the follow-up, other blood samples were collected from this patient and long-term CTC cultures could be established at the time of the second and third relapse (Fig. 1B). At the time of the second relapse, the new CTC line CTC-MCC-41.4 was derived using a blood sample that contained 3,278 CTCs/7.5 mL and 962 CTC clusters (<2 to 13 CTCs/microemboli). At the time of the third relapse, seven new CTC-MCC-41.5 lines were established from a blood sample with 286 CTCs/7.5 mL (but only 3 clusters of 2 CTCs/microemboli): CTC-MCC-41.5A, CTC-MCC-41.5B, CTC-MCC-41.5C, CTC-MCC-41.5D, CTC-MCC-41.5E, CTC-MCC-41.5F, and CTC-MCC-41.5G. Analysis of the correlation between the CTC number in patient 044’s blood samples and the successful establishment of CTC lines (Fig. 1C) suggests that, in colon cancer, around 300 CTCs are required to select metastasis-competent CTCs that can self-renew and grow *in vitro*. In addition, based on the CellSearch cell images, CTCs were present as single cells and clusters in the first samples, and as single cells in the last sample at the time of the third relapse (Fig. 1C). Moreover, in the first samples, giant and small CTCs could be detected simultaneously, whereas only very small (4–6 µm) and small (8 µm) CTCs were present in the last sample before the patient’s death.

### *In vitro* culture and morphological description of the nine colon CTC lines

**Figure 2:**
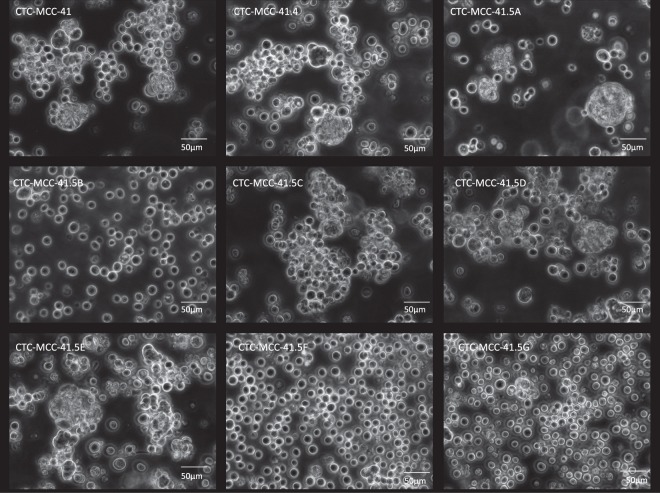
*In vitro* culture of the colon CTC lines. Representative images of each CTC line (Zeiss Axio Observer D1).

The CTC-MCC-41.4 line was obtained by pooling several CTCs that started to proliferate at the same time in all wells of the culture plate. This new CTC line showed isolated tumor cells with a mean diameter of 15 µm (50%) and colospheres with a size that could reach 100 µm (50%) (Fig. 2), as observed in the CTC-MCC-41 line.

In the CTC-MCC-41.5 lines, CTCs started to proliferate in different wells at different time points. Therefore, they were kept separated, giving rise to the different lines (CTC-MCC-41.5A, B, C, D, E, F and G). The first three CTC-MCC-41.5 lines (CTC-MCC-41.5D, CTC-MCC-41.5F and CTC-MCC-41.5G) appeared after 18 days of culture. They showed different morphologies and behaved differently. CTC-MCC-41.5D cells grew as isolated cells (50%) and also as big colospheres (up to 100 µm). The CTC-MCC-41.5F and CTC-MCC-41.5G lines were composed essentially of isolated cells (70%) and small colospheres (up to 50 µm) for the remaining 30%. After 24 days of culture, three additional CTC lines appeared: the CTC-MCC-41.5A line, characterized by the presence of isolated tumor cells and colospheres (50%-50%); the CTC-MCC-41.5B line, with 70% of small isolated cells and few small colospheres (30%); and the CTC-MCC-41.5C line, made mostly of large colospheres (80%, up to 200 µm) and few isolated cells (20%). Finally, after 34 days of culture, the last CTC line (CTC-MCC-41.5E) displayed isolated cells (50%) and big colospheres (50%) (Fig. 2).

### Characterization of the CTC lines

#### Genomic analysis

**Figure 3:**
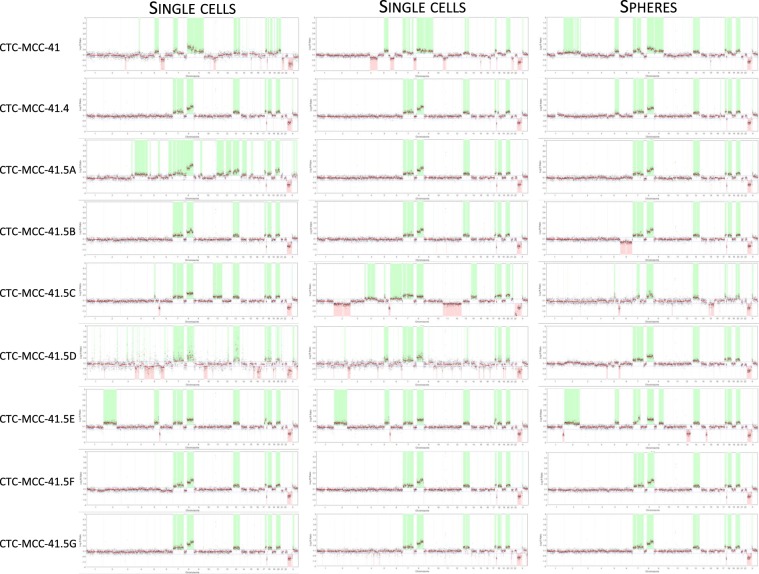
Copy number alterations in the nine CTC cell lines determined by next-generation sequencing. Chromosomal aberrations in the whole genome (x-axis) were investigated in two single-cell and one colosphere sample for each colon CTC line. Copy number gains are shaded green, and copy number losses are shaded red. Red horizontal lines represent the estimated copy number value (y-axis) and mark the break points between intrachromosomal copy-number alterations.

**Figure 4:**
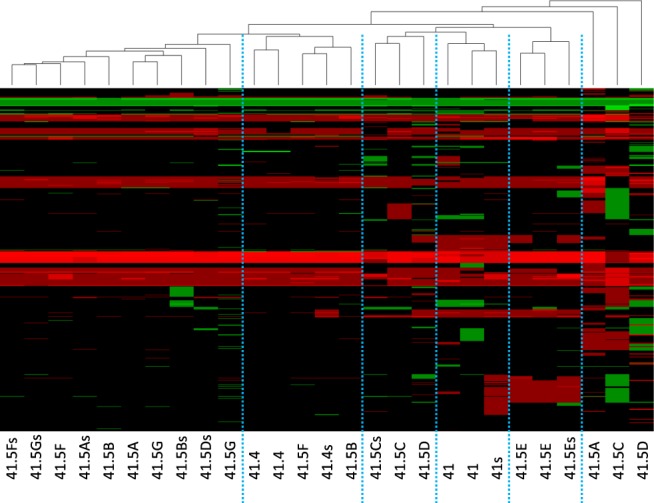
Hierarchical clustering of the nine colon CTC lines. Whole genome analysis results, from chromosome 1 to X (bottom to top), of two single-cell and one colosphere (s) sample for each colon CTC line allowed their subdivision in different clusters. Red represents gains, and green represents losses of genomic material.

To prove the tumor nature of the nine CTC-MCC-lines, copy number alterations (CNA) were assessed genome-wide in two single cells and one colosphere for each CTC line by next-generation sequencing (NGS). This analysis revealed a wide spectrum of chromosomal aberrations (Fig. 3). Typical colorectal cancer–related chromosomal gains and losses were found throughout the genome in all investigated samples, particularly gains in chromosomes 7, 8 and 20 and losses in chromosome 22. However, no specific CNA signature could be detected in the first CTC line (CTC-MCC-41), and in those established during disease progression (CTC-MCC-41.4 and -41.5A-G). Based on the phylogenetic analysis results using the copy number median ratios, samples (single cells and colospheres) could be clustered in six subgroups (Fig. 4). The largest group (on the left side on Fig. 4) contained all the CTC-MCC-41.5G samples, the colosphere sample and one single-cell sample from the CTC-MCC-41.5A, 5B and 5F lines, and the CTC-MCC-41.5D colosphere sample. These results demonstrate clearly the high genomic variability within of each colon CTC line, with the exception of the CTC-MCC-41 and −41.5E lines that clustered in two individual groups. Moreover, the absence of cell line-specific CNA signature suggests that CTC changes over time were not driven by additional CNAs and/or did not cause any new CNA.

Concerning specific mutations, all samples carried the *BRAF*^V600E^ mutation with a stable allele frequency of 50%, in agreement with a heterozygote mutation (Supplementary Table S1). *KRAS* was wild type (wt) in all CTC lines. This mutation profile (*BRAF*^mut^*KRAS*^wt^) mirrors the one detected in the primary tumor, lymph node metastasis, and also tumor xenografts from immunodeficient mice after injection of CTC-MCC-41 cells^13^.

#### Transcriptomic characterization

To characterize these CTC lines, the transcriptome profiles of the eight new colon CTC lines were compared with those of the first line (CTC-MCC-41) and of two commercial colon cancer cell lines (HT-29 and SW-620; derived from a primary tumor and from a lymph node metastasis, respectively). The panel of messenger RNAs (mRNAs) to be analyzed (Supplementary Table S2) was chosen based on literature data and the transcriptomic profile of the CTC-MCC-41 line^14^, and included genes encoding molecules involved in many tumor cell features (e.g., epithelial, mesenchymal, stemness, energetic metabolism and immune system escape).

**Figure 5:**
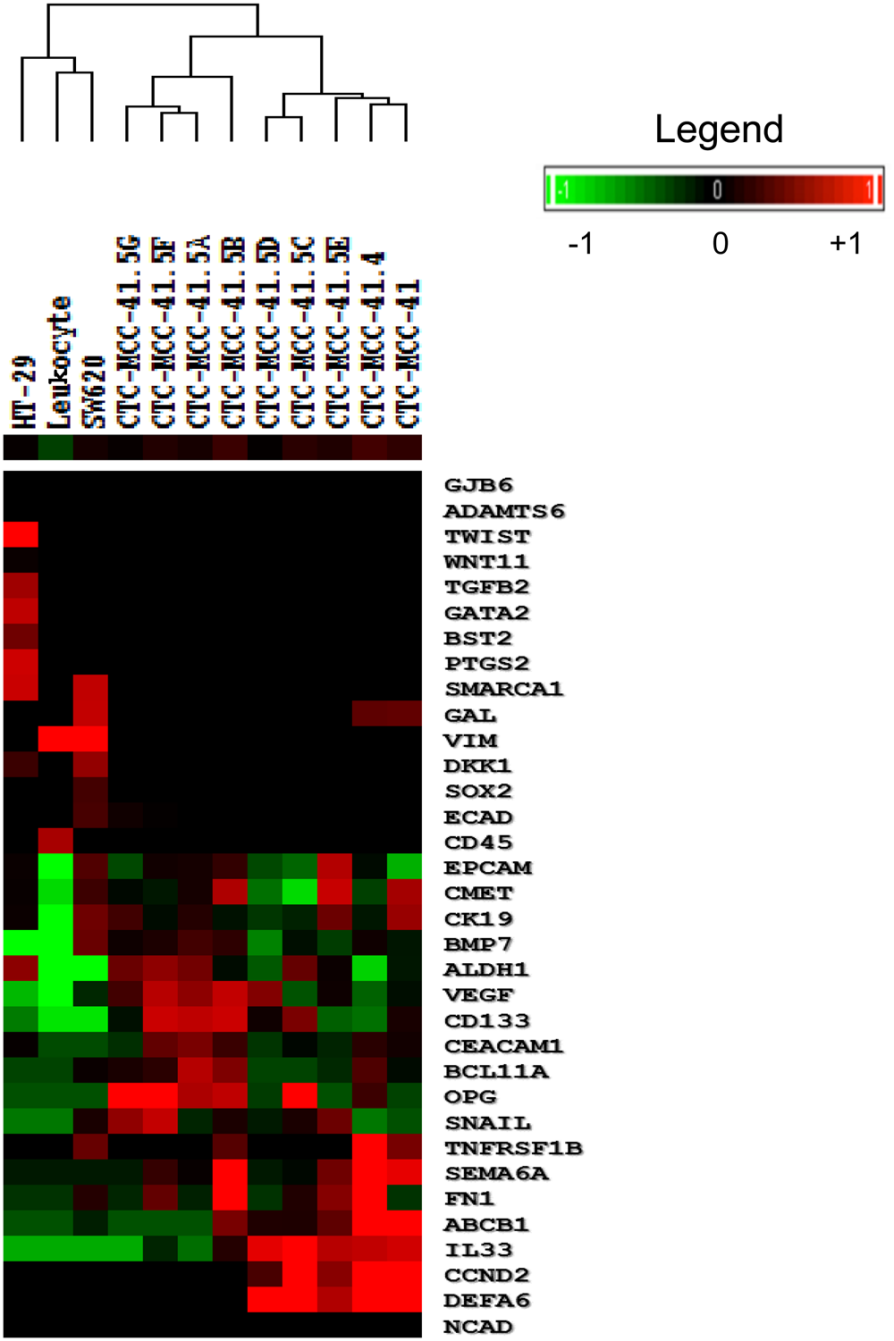
Hierarchical clustering of the nine colon CTC lines relative to the HT-29 and SW620 colorectal cancer cell lines and leukocytes. The expression of 34 mRNAs (Supplementary Table S2) was analyzed in the nine colon CTC lines, HT-29 cells (from a primary colorectal tumor), and SW620 cells (from a lymph node metastasis of colon cancer). These transcripts covered different properties of tumor cells: epithelial (*ECAD, CK19, EPCAM*), mesenchymal (*NCAD, VIM, FN1*), stemness (*CD133, ALDH1*), angiogenesis (*VEGF*), proto-oncogene (*MET*), osteomimicry (*OPG, BMP7*), EMT (*SNAIL, TWIST*, *WNT, DKK1*), immune system (*IL33*, *DEFA6*, *CEACAM1*), apoptosis (*CCND2, BCL11A, TNFRS1B*), DNA repair (*GAL, SMARCA1*), cell growth (*BST2, SEMA6A*), cell interactions (*ADAMTS6, GJB6, GATA2, TGFB2*) and energy metabolism (*ABCB1, PTGS2*). *B2M* (housekeeping gene) and *CD45* (leukocyte marker) were used as reference genes. Leukocyte cDNA was used as negative control. Results were normalized to the expression level of the reference *B2M* gene. Cell lines were clustered based on their expression profile.

The transcriptome data could be stratified in four different groups according to the expression of each mRNA in the CTC lines (Supplementary Table S3). The first group included genes with an identical expression profile (present/absent) in all the cell lines analyzed (colon CTC, HT-29 and SW-620). All cell lines expressed epithelial cell adhesion molecule (*EPCAM)*, cytokeratin 19 (*CK19)*, E-cadherin (*ECAD)*, *CMET*, carcinoembryonic antigen-related cell adhesion molecule 1 (*CEACAM1)*, vascular endothelial growth factor *(VEGF)*, snail family transcription repressor 1 (*SNAIL)* and aldehyde dehydrogenase 1 (*ALDH1)*, but not *CD45*. This confirmed the epithelial origin of all these cell lines. A second group included transcripts that were differentially expressed in the colon CTC lines and in HT-29 and SW620 cells: (i) interleukin 33 (*IL33*), cyclin D2 (*CCND2*), semaphorin 6A (*SEMA6A*) and osteoprotegerin (*OPG)* that were expressed only in the colon CTC lines; and (ii) SWI/SNF related, matrix associated, actin dependent regulator of chromatin, subfamily A, member 1 (*SMARCA1*) and dickkopf WNT signaling pathway inhibitor 1 (*DKK1*) that were expressed only in HT-29 and SW-620 cells. The third group included genes that (i) were expressed in the colon CTC lines and in SW620 cells: B-cell lymphoma 11A (*BCL11A*), tumor necrosis factor receptor superfamilly 1B (*TNFRS1B*), bone morphogenetic protein 7 (*BMP7*) and fibronectin 1 (*FN1*); and (ii) were not expressed in the CTC lines and SW620 cells: adam metallopeptidase with thrombospondin type 1 motif 6 (*ADAMTS6*), Gata binding protein 2 (*GATA2*), gap junction protein beta 6 (*GJB6*), transforming growth factor beta 2 (*TGFB2*), prostaglandin endoperoxidase synthase 2 (*PTGS2*), bone marrow stromal cell antigen 2 (*BST2*), Wnt family member 11 (*WNT11*) and *TWIST*. The last group included genes that were downregulated in all colon CTC lines and in HT-29 cells, but expressed in SW620 cells: SRY-related HMG-box gene 2 (*SOX2*) and vimentin (*VIM*). Hierarchical clustering analysis based on the mRNA expression profile clearly segregated the CTC lines from the HT-29 and SW-620 cell lines (Fig. 5). Moreover, the colon CTC lines could be clustered in several sub-groups based on their different expression profiles.

#### Proteomic characterization

**Figure 6:**
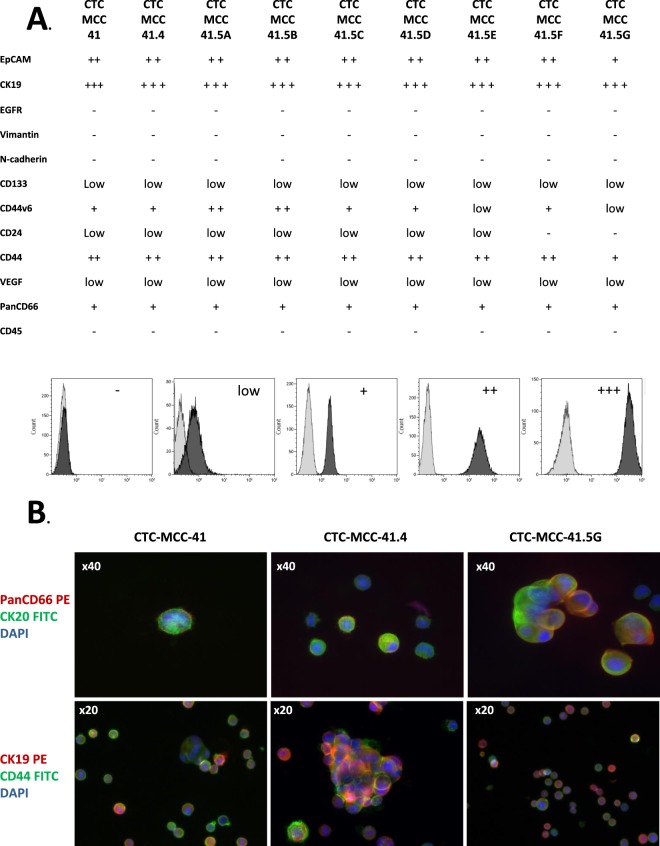
Protein expression analysis in the colon CTC lines. (**A**). Flow cytometry experiments. The expression of a set of proteins was analyzed using different fluorescent antibodies to characterize the phenotype of each CTC line: epithelial (EpCAM, CK19, EGFR), mesenchymal (vimentin, N-cadherin), stemness (CD133, CD44v6, CD44/CD24 ratio), angiogenic (VEGF), immune system escape (PanCD66) and hematopoietic (CD45). The histograms at the bottom show the expression levels (from negative to +++) used to describe the results. (**B**). Immunofluorescence experiments. Representative images of CTC-MCC-41, CTC-MCC-41.4 and CTC-MCC-41.5G single cells and colospheres showing co-expression of PanCD66 (CEACAM) and CK20, or CD44 and CK19. Nuclei were stained with DAPI (blue). PE, phycoerythrin; FITC, fluorescein isothiocyanate.

The expression profile of a large panel of proteins to cover different properties of colorectal tumor cells (e.g., epithelial-mesenchymal plasticity, stem-cell properties, immunomodulation capacity and angiogenesis) was assessed in the nine colon CTC lines by flow cytometry (Fig. 6A). As already reported for the CTC-MCC-41 line^13^, the new CTC lines also strongly expressed epithelial markers, such as EpCAM and cytokeratins (CK8, 18, 19 & 20), but none of the tested mesenchymal markers (N-cadherin and vimentin). In addition, the expression of CD133 and CD44v6 and the CD24^(low)^/CD44^(high)^ ratio indicated stem cell-like characteristics^15^. All CTC lines weakly expressed VEGF and PanCD66 (A, B, C & E), also known as CEACAM (1, 2, 3 and 5). The absence of CD45 expression confirmed that these cells were not leucocytes. Moreover, the eight new colon CTC lines did not express endothelial proteins (CD31 and CD105), hematopoietic progenitor markers (CD34) and C-X-C chemokine receptor type 4 (CXCR4), a receptor expressed on breast tumor cells and involved in the chemoattraction to specific organs^16^ (Supplementary Table S4). All the data obtained by flow cytometry were confirmed by concomitant immunofluorescence analysis (Fig. 6B with examples of co-expression of specific proteins).

#### Functional characterization

**Figure 7:**
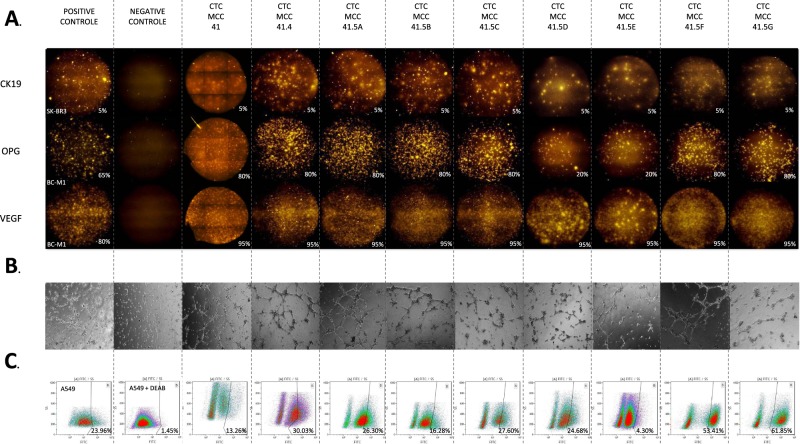
Functional analysis of the colon CTC lines. (**A**) Representative images of the EPISPOT results. Secretion, release and shedding of CK19 (upper), OPG (middle) and VEGF (lower panels) by viable CTCs were assessed using the fluoro-EPISPOT assay (C.T.L. Elispot reader). The breast cancer cell lines SKBR3 and BC-M1 were used as positive controls for CK19, and for OPG and VEGF, respectively. The negative control well was without cells (magnification X5). Each fluorescent immunospot corresponds to the protein fingerprint of one secreting cell. (**B**) Endothelial cell tube formation assay. Endothelial cell tube formation was observed after 6 h (representative images; magnification X5) of cultures with endothelial cell medium (positive control), basal medium (RPMI 1640 alone; negative control), and supernatant from the nine CTC lines cultured in basal medium for 48 h. (**C**) ALDH activity assay by flow cytometry. Gates for positive signal were designed for each CTC line based on the negative control performed with the DEAB reagent. The lung cancer cell line A549 was used as positive control. The percentage of ALDH-positive cells is indicated for each condition.

Different functional assays were then performed to assess the biology of these tumor cells: EPISPOT assay, *in vitro* endothelial cell tube formation assay, and ALDH1 activity assay (Fig. 7).

The fluoro-EPISPOT assay assesses the ability of viable tumor cells to secrete, shed or release specific proteins. The fluoro-EPISPOT assay results indicated that CTC-MCC-41, CTC-MCC-41.4 and all the CTC-MCC-41.5 lines could release CK19 (5% of cells), and secrete VEGF (95% of cells) (Fig. 7A, upper and lower panels). Moreover, about 80% of CTC-MCC-41.4, CTC-MCC-41.5A, CTC-MCC-41.5B, CTC-MCC-41.5C, CTC-MCC-41.5F and CTC-MCC-41.5G cells could actively shed OPG, whereas only 20% of CTC-MCC-41.5D and CTC-MCC-41.E cells showed OPG immunospots (Fig. 7A, middle panels).

As the colon CTC lines expressed and secreted VEGF, *in vitro* endothelial cell tube formation was also tested (Fig. 7B). Endothelial cell tubes were observed only when endothelial cells were cultured in endothelial cell medium for 6 hours (positive control), but not in the presence of basal medium (RPMI 1640 alone; negative control). Supernatants of all colon CTC lines also could induce endothelial cell tube formation, with a higher number of tube-like structures than the positive control for the CTC-MCC-41, CTC-MCC-41.4, CTC-MCC-41.5A, CTC-MCC-41.5B and CTC-MCC-41.5F cell supernatants (Fig. 7B).

Elevated ALDH activity is an important biomarker of stem and progenitor cells in epithelial cancers^17^. The ALDH activity assay (Fig. 7C) highlighted the presence of a subpopulation of ALDH-positive cells in all CTC lines, but for CTC-MCC-41.5E cells only 4.30% were ALDH-positive. CTC-MCC-41.5F and CTC-MCC-41.5G cells displayed the highest percentages of ALDH-positive cells (53.41% and 61.85%, respectively).

## Discussion

Subsets of cancer cells can disseminate in the body to a distant site where they colonize the new organ and form metastases^18^. Some of these CTCs have a cancer stem-cell phenotype, as indicated by different studies in breast, lung, prostate and colon cancer^10,11,13,19–21^.

In 2015, our group published the first experimental proof that a stable colon CTC line (CTC-MCC-41) could be established from CTCs isolated from the blood of a patient with metastatic colon cancer^13^. The present study provides the characterization of the other eight CTC lines derived from CTCs isolated from the same patient at different times during his follow-up before/after treatment and disease progression. This unique material will allow assessing the clonal selection of aggressive metastasis-competent cancer cells during cancer progression.

Our previous^13^ and present data on the establishment of colon CTC lines suggest that a CTC count of approximately 300 (using the CellSearch system) in blood samples from patients with colon cancer is a prerequisite for successful CTC expansion in culture. However, the number of CTCs is lower in the blood of patients with colorectal cancer that with other cancer types. In patients with colon cancer, viable CTCs can be captured in mesenteric blood samples, indicating that many of them are trapped in the liver^22^. Furthermore, this specific patient had the BRAF mutation, which is relatively rare (about 10%) and highly aggressive in CRC^23^. Indeed, only 8 out of 95 patients were positive for this rare colorectal mutation. Interestingly, no correlation between the BRAF mutation and a high number of CTCs has been observed in the COLOSPOT study. All these data clearly show how challenging it is to establish a CTC line from patients with colon cancer even with distant metastases, and may explain why no CTC culture or cell line has been reported in the literature, with the exception by our group.

To verify that these cell lines are of cancer origin, we performed genotyping analyses. CNA analysis by NGS demonstrated that these CTC lines were cancer cell lines with cancer–specific genomic aberrations. This analysis highlighted CNA differences between samples (colosphere and single cells) from the same CTC line and also between lines, indicating that these lines have a polyclonal origin. However, we did not observe any CNA associated with disease progression. Indeed, the first CTC line (obtained at diagnosis) and the other lines (obtained at different time points after treatment) were all highly heterogeneous at the inter- and intra-line level. This suggests that therapy did not cause any significant change in the cancer CNA signature. However, we cannot exclude therapy-driven selection of resistance-conferring mutations, other than *BRAF* mutations that were detected with a stable frequency in all CTC lines.

Visual monitoring of the cultures showed again a clear intra- and inter-line heterogeneity: colospheres as well as small and big single cancer cells. This suggests the absence of a preferred morphology for these tumor cells, consistent with the observation that both small and large CTCs are linked to unfavorable prognosis^24^. Recent studies have emphasized that CTC clusters may have an increased malignant capacity^25^, and microemboli were observed in the blood samples of this patient with metastatic colon cancer during his follow-up.

Epithelial-to-mesenchymal transition (EMT) is a complex process that supports the migratory capacity of epithelial tumor cells. EMT also plays a crucial role in promoting cancer metastasis by various mechanisms, including CTC release from the primary tumor and distant metastases^26^. All nine CTC lines presented a stable epithelial phenotype, as indicated by the expression of epithelial markers, such as EPCAM and cytokeratins (8, 18, 19 & 20). Concerning markers of mesenchymal transition, all nine CTC lines expressed *FN1* and *SNAIL*, but not vimentin or N-cadherin (mRNA and protein). Moreover, in all CTC cell lines, *ECAD* was weakly expressed and *ADAMTS6*, *GATA2*, *GJB6*, *TGFB2*, *PTGS2* and *BST2*, genes encoding cell-cell junctions, focal adhesion or cytoskeletal proteins, were clearly downregulated^14^. This is in accordance with CTC loss of adherent cell properties to leave the tumor and reach the bloodstream. These data indicate that these colon CTC lines are tumor cells with an intermediate EMT phenotype. The acquisition of mesenchymal features by CTCs could reveal some plasticity required for dissemination to distant organs^26^. In addition, the expression of *TNFRSF1B* and *BCL11A*, linked to apoptosis suppression^27^, could indicate another CTC hallmark called «anoikis resistance», a mechanism to avoid cell death when tumor cells are not attached, but swim in the bloodstream^18^.

Stemness features, such as expression of CD133, CD44v6 (a colorectal stemness marker)^15^, *BMP7*, *GAL* and *SEMA6A*, and the CD44^high^/CD24^low^ ratio^28^, were detected in all nine CTC lines. Moreover, they all showed ALDH activity, a well-known stem cell marker^17^. The combination of EMT phenotype and stem-cell characteristics should favor their dissemination and ability to form distant metastases^29^.

In addition, the expression of *OPG*, *BMP7* and *TNFRSF1B* (involved in osteogenic activity) suggests that these CTCs have the ability to lodge in the bone marrow. However, this patient developed liver metastases, without any evidence of bone metastases. Therefore, we hypothesize that these CTCs could have been released from the bone marrow where they were preserved in special niches as dormant tumor cells with the ability to mimic osteogenic cells.

Concerning the immune system escape and survival, the PD-1/PD-L1 axis has been extensively studied in the last years after the introduction of cancer immunotherapy. Recently, we demonstrated that CTCs in breast cancer can express PD-L1 as a camouflage against T-lymphocytes^30^. However, PD-L1 expression was not detected in any of the nine CTC lines from this patient with colon cancer. Indeed, PD-L1 expression can vary, and it is still debated whether this protein is expressed more at early^31^ or late, metastatic stages^32,33^. Conversely, *CEACAM1, DEFA6* and *IL33*, three genes encoding proteins also involved in immune escape, were expressed by all nine CTC lines. *CEACAM1* upregulation at advanced stages of colorectal cancer is associated with invasiveness, metastasis, tumor progression and escape from the immune system^34^. Upregulation of *DEFA6*^14^ and *IL33*^35^ also is involved in immune mechanisms. Therefore, these three factors could be attractive targets for immunotherapy.

Another important feature of aggressive cancer cells is the capacity to induce neo-angiogenesis that is required to import nutrients and oxygen in the growing tumor. All the new CTC lines expressed and secreted VEGF, a key inducer of angiogenesis, as already shown for the CTC-MCC-41 line^13^. Moreover, a functional test based on *in vitro* tube formation using endothelial cells showed that CTC-MCC-41, 41.4, 41.5B, 41.5E and 41.5F cell supernatants induced a denser network of endothelial tubes than those of CTC-MCC-41.5C, D and G cells. These data highlight again the heterogeneity of the nine CTC lines. As the fluoro-EPISPOT assay showed that VEGF secretion was comparable in all nine CTC lines, other factors must be involved in CTC-induced angiogenesis. For instance, semaphorin 6A, a member of the semaphorin family involved in cell motility and proliferation, also contributes to vascular development and particularly to angiogenesis by modulating VEGFR2 signaling in endothelial cells^36^. The stronger *SEMA6A* expression in the CTC-MCC-41, 41.4, 41.5B, 41.5E and 41.5F lines could explain the heterogeneous results of the functional assay. Moreover, Loria *et al*. showed that forced *SEMA6A* overexpression induces anchorage-independent growth and significantly increases invasiveness. Interestingly, *SEMA6A* is overexpressed in *BRAF*^V600E^ melanoma cells^36^. This *BRAF* mutation was detected (*i*) in the primary tumor, (*ii*) in all the CTC lines, (*iii*) the lymph node metastasis, and also (*iv*) in tumor xenografts obtained by injection of CTC-MCC-41 cells^13^. Thus, *SEMA6A* might represent a new potential therapeutic target in *BRAF*^V600E^ cancers.

In conclusion, we established nine permanent colon CTC lines from the same patient with metastatic colon cancer at different time points during disease progression. We then thoroughly characterized these CTC lines, highlighting their heterogeneity, which suggests that they are derived from different clones, and also their common features. Indeed, they all show (i) an intermediate epithelial/mesenchymal phenotype, (ii) stem-cell like characteristics, (iii) the potential to induce angiogenesis, (iv) an osteomimetic signature, and (v) the capacity to escape from the immune system. These features suggest that they could initiate metastases at distant sites. Moreover, in these CTC lines, we observed changes in RNA and protein expression, while the analysis of chromosomal CNA revealed no significant pattern of selected genomic alterations. For instance, the stem-cell like characteristics progressively increased in the CTC lines isolated at later stages of cancer progression. These data indicate that CTCs cultured from sequential liquid biopsies during therapy have common traits, but that treatment-resistant clones with distinct phenotypic characteristics are selected.

## Methods

### Patients and blood collection

After signature of the informed consent, peripheral blood samples from patients with non-resectable metastatic colorectal adenocarcinoma were collected before the start and during first-line chemotherapy (FOLFIRI and bevacizumab) in the framework of the COLOSPOT study (NCT01596790; 168 patients included between 2011 and 2015). The study was carried out in accordance with the World Medical Association Declaration of Helsinki and the guidelines for experimentation with humans by the Bioethical committee “Sud Méditerranée III”. The experimental protocol was approved (Approval reference No. 2011-A01130-41) by the Bioethical committee “Sud Méditerranée III”. All included subjects provided written informed consent for participation in this study and the publication of results. The Blood (10 mL) was collected in CellSave tubes (SILICON BIOSYSTEMS, Menarini, Bologna, Italy) for CTC enumeration and in EDTA tubes (10 mL) for *in vitro* CTC culture.

### Clinical characteristics and history of patient 044

Patient 044 was diagnosed at an advanced stage of colon cancer (lymph nodes and liver metastases, but no evidence of bone metastasis). He showed good tolerance to the first-line treatment (FOLFIRI + bevacizumab), but, after 5 cycles, had to stop it because of cancer progression (RECIST criteria). A second-line treatment was immediately initiated (FOLFOX + bevacizumab), but again, the treatment was stopped after three cycles due to clinical progression (peritoneal carcinomatosis), and no other treatment was planned. The patient was hospitalized for abdominal pain and occlusive syndrome, and a new relapse was diagnosed. He died 6 months after the diagnosis of cancer, 2 months after the last treatment, and 1 week after the last blood sample collection. Additional clinical data about this special patient are available in the Supplementary Table S5.

### CTC enrichment and *in vitro* culture

The blood collected in EDTA tubes was immediately used for CTC culture. First, viable CTCs were enriched through depletion of hematopoietic CD45^(+)^ cells using the RosetteSep Human Circulating Epithelial Tumor Cell Enrichment Cocktail (STEMCELL TECHNOLOGIES, Vancouver, Canada), according to the manufacturer’s protocol. Then, CD45^(−)^ CTCs were incubated at 37 °C in 24-well non-adherent plates with CTC culture medium (RPMI1640 with EGF and FGF-2, Insulin-Transferrin-Selenium supplement, L-Glutamine), as previously described^13^. Colospheres and tumor cells were observed during the first weeks of culture and were then transferred to new 24-well plates for further growth and to T25 flasks for cell expansion. In these conditions, CTCs could be quickly expanded and after a few months, billions of tumor cells were obtained.

### Cell culture

The colorectal cell lines HT-29 (ATCC HTB-38) and SW620 (ATCC CCL-227) were used as control for several tests, as well as the lung cancer cell line A549 (ATCC CRM-CCL-185), the mammary cancer cell lines SKBR3 (ATCC HTB-30) and BC-M1 (an established breast cancer cell line from a single disseminated tumor cell in the bone marrow of a patient with non-metastatic breast cancer patient)^37^. HT-29 and SKBR3 cells were maintained in Dulbecco’s Modified Eagle’s medium (DMEM), 10% fetal bovine serum (FBS), and 1% penicillin/streptomycin. A549 cells were maintained in DMEM supplemented with 10 mM HEPES, 10% FBS and 1% penicillin/streptomycin. SW620 cells were maintained in RPMI 1640 with L-glutamine and 10% FBS, and the colon CTC lines and BC-M1 cells in CTC culture medium. Endothelial cells were cultured in M199 (M4530, SIGMA ALDRICH, St Louis, USA) containing 0.4 ng/ml endothelial growth factors (βECGF; E1388, SIGMA ALDRICH), 20 µg/ml heparin (H4784, SIGMA ALDRICH, Saint-Louis, USA), 1% penicillin/streptomycin and 20% FBS (endothelial cell medium).

### Next-generation sequencing for copy-number alterations

One colosphere and two single cells were transferred to individual PCR tubes (0.2 mL) for whole genome amplification using the PicoPlex WGA Kit, according to the manufacturer’s protocol (RUBICON GENOMICS, Ann Arbor, USA). Next, libraries were prepared using the TruSeq DNA Sample Prep Kit according to the manufacturer’s protocol. NGS was performed on a HiSeq. 2500 apparatus (ILLUMINA, San Diego, USA) using single reads for 100 cycles. Sequence analyses and CNA identification were performed using Control-FREEC and a custom-made script in MatLab.

### CTC mutational status

DNA was extracted from the nine colon CTC lines using the QIAamp DNA Mini Kit (QIAGEN GmbH, Hilden, Germany), according to the manufacturer’s instructions. DNA yield and purity were assessed with a Qubit spectrophotometer (THERMO FISHER SCIENTIFIC, Wilmington, DE). DNA was stored at −20 °C in 10 mmol/L Tris and 0.5 mmol/L EDTA (pH 7.6).

Genomic DNA samples were genotyped using MassArray iPlex Pro (AGENA BIOSCIENCE), a Matrix Assisted Laser Desorption Ionization Time of Flight Mass Spectrometry assay. A specific colorectal cancer panel (Imagenome Labosud, France) was customized for the detection of mutations in six oncogenes (Supplementary Table S6), selected on the basis of their role in colorectal cancer diagnosis, treatment and follow-up. The Assay Design Suite software (ADS, AGENA BIOSCIENCE) was used to design four sets of PCRs and iPLEX primers. Briefly, 2 µL (10 ng) of genomic DNA was amplified in 4 wells by multiplex PCR with specific primers for wild type (WT) and mutant DNA (mtDNA), followed by shrimp-alkaline-phosphatase (SAP) treatment to remove the surplus nucleotides. Then, a primer single base extension reaction (iPLEX Pro) was performed with mass-modified ddNTP terminators. The extension products were dispensed on a SpectroCHIP Array and detected by MassARRAY mass spectrometry. Data were analyzed with the MassARRAY Typer Analyzer software^38^.

### RT-qPCR analysis of colon CTC lines

Messenger RNA (mRNA) was extracted from each CTC line using the RNeasy Mini Kit (74106, QIAGEN, Hilden, Germany), and complementary DNA (cDNA) was obtained by reverse transcription (RT) using the SuperScript III First-Strand Synthesis Super Mix kit (18080, INVITROGEN, Carlsbad, USA), according to the manufacturer’s instructions. Semi-quantitative PCR that targeted a wide range of sequences (Supplementary Table S2) was used to assess the expression of a large panel of genes, including all the transcripts previously validated as up- or down-regulated after a microarray assay in the CTC-MCC-41 line^14^. In all experiments, β2-microglobulin (*B2M*) was used as reference housekeeping gene. One µL of each RT product was added to 9 µL of mix containing the two primers (6 µM each) and SYBR Green (600882, Brilliant III Ultra-Fast SYBR Green Master Mix, AGILENT TECHNOLOGIES, Santa Clara, USA). PCR reactions were performed on a QuantStudio 5 real-time PCR instrument (THERMO FISHER, Waltham, USA) as follows: 95 °C for 3 min, followed by 40 cycles of 95 °C for 5 s, 60 °C for 10 s, and then 1 cycle at 95 °C for 1 min, 55 °C for 5 s, 95 °C for 30 s, 37 °C for 30 s. Results were analyzed using the QuantStudio Design and Analysis Software. Hierarchical clustering was computed with the Cluster and Treeview software packages (PMID: 9843981).

### Flow cytometry and immunofluorescence experiments

A panel of antibodies (Supplementary Table S7) was used to characterize the nine colon CTC lines. The IntraPrep Permeabilization Reagent kit (A07803, Beckman Coulter, Brea, USA) was employed to study the expression of intracellular proteins, whereas conjugated antibodies were directly used for membrane proteins.

Labeled tumor cells were analyzed using a Cyan cytometer (BECKMAN COULTER) and data interpreted with the Kaluza software (BECKMAN COULTER). In parallel, labeled tumor cells were also loaded on glass slides, using a cytospin, and observed under a fluorescent microscope (Axio Observer D1, ZEISS, Oberkochen, Germany).

### Fluoro-EPISPOT assays

The fluoro-EPISPOT assays for CK19, VEGF and OPG were performed as previously described^13,39,40^.

### Endothelial cell tube formation assay

24-well plates were coated with 250 µL/well Matrigel (BD BIOSCIENCES, San Jose, USA) and left at 37 °C for 30 min. Endothelial cells (10^5^ cells/well) were then seeded and cultured for 6 h with different media: RPMI 1640 alone (basal medium; negative control), endothelial cell medium with 20% FBS (positive control), and supernatant from the CTC lines cultured with basal medium for 48 h.

### ALDH1 activity assay

ALDH1 activity was measured using the ALDEFLUOR kit (01700 – STEMCELL TECHNOLOGIES) following the manufacturer’s instructions. Briefly, 5 µL of ALDEFLUOR Reagent was added to the cell suspension and half of the cell mixture was immediately transferred to another tube with the ALDEFLUOR DEAB Reagent (an ALDH activity inhibitor) to serve as internal negative control to quantify ALDH activity in each cell line. A549 cells were used as positive control, as recommended by the manufacturer.

### Statement of Significance

Only metastasis-competent CTCs lead to cancer relapse, but their specific signature is not known. The nine sequential autologous CTC lines we derived from a patient with metastatic colorectal cancer will help to study CTC changes during cancer progression, a prerequisite to discover new therapeutic targets and to propose personalized treatments.

## Electronic supplementary material

Supplementary information
