# Supplementary material for: Autologous cell lines from circulating colon cancer cells captured from sequential liquid biopsies as model to study therapy-driven tumor changes
Source: Sci Rep. 2018 Oct 29;8:15931. doi: 10.1038/s41598-018-34365-z (PMC6206091; doi:10.1038/s41598-018-34365-z)

Autologous cell lines from circulating colon cancer cells captured from sequential liquid biopsies as model to study therapy-driven tumor changes

Alexandra Soler[1*], Laure Cayrefourcq[1*], Thibault Mazard[2], Anna Babayan[3], Pierre-Jean Lamy[4], Said Assou[5], Eric Assenat[6], Klaus Pantel[3], Catherine Alix-Panabières[1].

*contributed equally to this article

[1]Laboratory of Rare Human Circulating Cells, University Medical Centre of Montpellier, EA2415, Montpellier, France;

[2]Department of Medical Oncology, Cancer Institute of Montpellier, Montpellier, France;

[3]Department of Tumor Biology, University Medical Centre Hamburg-Eppendorf, Hamburg, Germany;

[4]Imagenome, Labosud, Montpellier, France;

[5]IRMB, Montpellier University, INSERM U1183, University Medical Centre of Montpellier, Montpellier, F34000 France;

[6]Department of Medical Oncology, University Medical Centre of Montpellier, Montpellier, France.

Supplementary Table S1. Oncogene mutations analyzed by Imagenome™ colorectal cancer panel.

	Exons	Codons
KRAS	2	12, 13
	3	61
	4	146
NRAS	2	12, 13
	3	61
	4	146
BRAF	15	600
PIK3CA	9	539, 542, 545, 546
	20	1047, 1048, 1049
PTEN	7	159, 233, 267 (del)
AKT	4	17

Supplementary Table S2. Sequence of the forward and reverse primers used for qRT-PCR analysis

Gene	Entrez Gene Name	Location	Type	Oligo Name	Sequence 5' to 3'	
DEFA6	Defensin alpha 6	Extracellular Space	Other	Forward Sequence	ATGACCAGGACTTTGCCGTCTC	NM_001926
				Reverse Sequence	CATGACAGTGCAGGTCCCATAG	
BCL11A	B-cell CLL/lymphoma 11A	Nucleus	Transcription regulator	Forward Sequence	CTCGTTCTGCACATGGAGCTCT	NM_022893
				Reverse Sequence	GCAAGAGAAACCATGCACTGGTG	
IL33	Interleukin 33	Extracellular Space	Cytokine	Forward Sequence	GCCTGTCAACAGCAGTCTACTG	NM_033439
				Reverse Sequence	TGTGCTTAGAGAAGCAAGATACTC	
HS6ST2	Heparan sulfate 6-O-sulfotransferase 2	Plasma Membrane	Enzyme	Forward Sequence	CTCTCCGTCATCCACAAAGACC	NM_001077188
				Reverse Sequence	CAGGGATGCTTTCCATGTTGCC	
GJB6	Gap junction protein beta 6	Plasma Membrane	Transporter	Forward Sequence	GAAACCACTCGCAAGTTCAGGC	NM_006783
				Reverse Sequence	AGGCTGCTTCAAAGATGATTCGG	
SMARCA1	SWI/SNF related, matrix associated, actin dependent regulator of chromatin, subfamily a, member 1	Nucleus	Transcription regulator	Forward Sequence	AACCTGGCAAGTGCTGATGTGG	NM_003069
				Reverse Sequence	CAGTGTTGTCAGTGATGAGACGG	
ADAMTS6	ADAM metallopeptidase with thrombospondin type 1 motif 6	Extracellular Space	Peptidase	Forward Sequence	GTGATCCTGACAGTAAGCCACC	NM_197941
				Reverse Sequence	CCACCATCACAAGTCTTGCTGC	
BMP7	Bone morphogenetic protein 7	Extracellular Space	Growth factor	Forward Sequence	GAGTGTGCCTTCCCTCTGAACT	NM_001719
				Reverse Sequence	AGGACGGAGATGGCATTGAGCT	
ABCB1	ATP binding cassette subfamily B member 1	Plasma Membrane	Transporter	Forward Sequence	GCTGTCAAGGAAGCCAATGCCT	NM_000927
				Reverse Sequence	TGCAATGGCGATCCTCTGCTTC	
CCND2	Cyclin D2	Nucleus	Other	Forward Sequence	GAGAAGCTGTCTCTGATCCGCA	NM_001759
				Reverse Sequence	CTTCCAGTTGCGATCATCGACG	
TNFRSF1B	TNF receptor superfamily member 1b	Plasma Membrane	Transmembrane receptor	Forward Sequence	CGTTCTCCAACACGACTTCATCC	NM_001066
				Reverse Sequence	ACGTGCAGACTGCATCCATGCT	
FN1	Fibronectin 1	Extracellular Space	Enzyme	Forward Sequence	ACAACACCGAGGTGACTGAGAC	NM_212482
				Reverse Sequence	GGACACAACGATGCTTCCTGAG	
SEMA6A	Semaphorin 6A	Plasma Membrane	Transmembrane receptor	Forward Sequence	ACCTGTATTGCCTCCAGAGACC	NM_020796
				Reverse Sequence	CCAGACCATCTGTATTGCCACG	
INHBB	Inhibin beta B subunit	Extracellular Space	Growth factor	Forward Sequence	GAAATCATCAGCTTCGCCGAGAC	NM_002193
				Reverse Sequence	GGCAGGAGTTTCAGGTAAAGCC	
GAL	Galanin and GMAP prepropeptide	Extracellular Space	Other	Forward Sequence	TGGCAACCACAGGTCATTCAGC	NM_015973
				Reverse Sequence	TCAGGTATGGACCTGTCAAAGCT	
PTGS2	Prostaglandin-endoperoxide synthase 2	Cytoplasm	Enzyme	Forward Sequence	CGGTGAAACTCTGGCTAGACAG	NM_000963
				Reverse Sequence	GCAAACCGTAGATGCTCAGGGA	
WNT11	Wnt family member 11	Extracellular Space	Other	Forward Sequence	CTGTGAAGGACTCGGAACTCGT	NM_004626
				Reverse Sequence	AGCTGTCGCTTCCGTTGGATGT	
TGFB2	Transforming growth factor beta 2	Extracellular Space	Growth factor	Forward Sequence	AAGAAGCGTGCTTTGGATGCGG	NM_003238
				Reverse Sequence	ATGCTCCAGCACAGAAGTTGGC	
DKK1	Dickkopf WNT signaling pathway inhibitor 1	Extracellular Space	Growth factor	Forward Sequence	GGTATTCCAGAAGAACCACCTTG	NM_012242
				Reverse Sequence	CTTGGACCAGAAGTGTCTAGCAC	
BST2	Bone marrow stromal cell antigen 2	Plasma Membrane	Other	Forward Sequence	TCTCCTGCAACAAGAGCTGACC	NM_004335
				Reverse Sequence	TCTCTGCATCCAGGGAAGCCAT	
GATA2	GATA binding protein 2	Nucleus	Transcription regulator	Forward Sequence	CAGCAAGGCTCGTTCCTGTTCA	NM_032638
				Reverse Sequence	ATGAGTGGTCGGTTCTGCCCAT	
EPCAM	Epithelial cell adhesion molecule	Plasma Membrane	Cell adhesion	Forward Sequence	ACAAGGACACTGAAATAACCTGC	NM_002354
				Reverse Sequence	TTTTGAGAAGAATTTTGAACCAGAT	
CK19	Keratin 19	Cytoplasm	Intermediate filament	Forward Sequence	GCGACTACAGCCACTACTACACG	NM_002276
				Reverse Sequence	CTTCGCATGTCACTCAGGATCT	
ECAD	Cadherin-1	Plasma Membrane	Cell adhesion	Forward Sequence	CCGAGAGCTACACGTTC	NM_004360
				Reverse Sequence	TCTTCAAAATTCACTCTGCC	
NCAD	Cadherin-2	Plasma Membrane	Cell adhesion	Forward Sequence	ACATATGTGATGACCGTAAC	NM_001792
				Reverse Sequence	TTTTTCTCGATCAAGTCCAG	
VIM	Vimentin	Cytoplasm	Intermediate filament	Forward Sequence	GGAAACTAATCTGGATTCACTC	NM_003380
				Reverse Sequence	CACTTCTAGTTTCAACCGTC	
OPG	TNF receptor superfamily member 11b	Plasma Membrane	Transmembrane receptor	Forward Sequence	GGTCTCCTGCTAACTCAGAAAGG	NM_002546
				Reverse Sequence	CAGCAAACCTGAAGAATGCCTCC	
VEGF	Vascular endothelial growth factor A	Extracellular Space	Growth factor	Forward Sequence	ATCTTCAAGCATCCTGTGT	NM_001025366.2
				Reverse Sequence	GCTCACCGCCTCGGCTTGT	
ALDH1	Aldehyde dehydrogenase 1 family member A1	Cytoplasm	Enzyme	Forward Sequence	CGGGAAAAGCAATCTGAAGAGGG	NM_000689
				Reverse Sequence	GATGCGGCTATACAACACTGGC	
CD133	Prominin-1	Plasma Membrane	Other	Forward Sequence	TTGAATGAAACTCCAGAGCAAA	NM_001145847
				Reverse Sequence	CCTCCTAGCACTGAATTGATACTG	
SOX2	SRY-box 2	Nucleus	Transcription regulator	Forward Sequence	AGCATGGAGAAAACCCGGTA	NM_003106
				Reverse Sequence	TTTTGCGTGAGTGTGGATGG	
TWIST	Twist-related protein	Nucleus	Transcription factor	Forward Sequence	GGCCGGAGACCTAGATGT	NM_000474
				Reverse Sequence	CACGCCCTGTTTCTTTGAAT	
SNAIL	Snail family transcriptional repressor	Nucleus	Transcription repressor	Forward Sequence	TGCCCTCAAGATGCACATCCGA	NM_005985
				Reverse Sequence	GGGACAGGAGAAGGGCTTCTC	
CEACAM1	Carcinoembryonic antigen related cell adhesion molecule 1	Plasma Membrane	Transporter	Forward Sequence	GAGTAGTGGCCCTGGTTGCTC	NM_001712
				Reverse Sequence	CGCTGGTCGCTTGCCCT	
CMET	MET proto-oncogene, receptor tyrosine kinase	Plasma Membrane	Transmembrane receptor	Forward Sequence	TTTATTAGTGGTGGGAGCACAAT	NM_001127500
				Reverse Sequence	GATGTTGACATGCCACTGTAAAG	
CD45	Protein tyrosine phosphatase, receptor type C	Plasma Membrane	Transmembrane receptor	Forward Sequence	TCACTTGCTCCTCAAACTGAGA	NM_002838
				Reverse Sequence	AGATGTACTGGGCCTCTACTTGA	
B2M	Beta-2-microglobulin	Plasma Membrane	Housekeeping gene	Forward Sequence	GTCTTTCAGCAAGGACTGGTCT	NM_004048
				Reverse Sequence	TTACATGTCTCGATCCCACTTAAC	

Supplementary Table S3. List of antibodies used for the CTC lines protein characterization.

Antibodies	Supplier	Clone	Ref	Conjugate	IgG
**CD105**	Miltenyi	43A4E1	130-094-941	PE	Mouse IgG1
**CD133**	Miltenyi	AC133	130-080-801	PE	Mouse IgG1
**CD24**	Miltenyi	32D12	130-095-953	PE	Mouse IgG1
**PanCD66 (a,c,d,e)**	Miltenyi	REA428	130-106-344	PE	Recombinant Human IgG1
**PanCK (8,18,19)**	Miltenyi	CK3-6H5	130-080-101	FITC	Mouse IgG1
**EpCAM**	Miltenyi	HEA-125	130-080-301	FITC	Mouse IgG1
**CD146**	Miltenyi	541-10B2	130-092-849	APC	Mouse IgG1
**E-Cadherin**	Miltenyi	67A4	130-095-412	APC	Mouse IgG1
**CD44**	Beckman Coulter	J.173	IM1219U	FITC	Mouse IgG1
**CD45**	Beckman Coulter	J.33	A07782	FITC	Mouse IgG1
**CD34**	Beckman Coulter	581	IM2709U	ECD	Mouse IgG1
**CD309**	Beckman Coulter	KDR-1	A64616	PC7	Mouse IgG1
**CXCR-4**	Beckman Coulter	12G5	A07409	PE	Mouse IgG2
**CD31**	Biolegend	WM59	303114	PB	Mouse IgG1
**EGFR**	Biolegend	AY13	352908	FITC	Mouse IgG1
**N-cadherin**	Biolegend	8C11	350805	PE	Mouse IgG1
**CK19**	Abcam	EP1508Y	Ab192643	A488	IgG
**ALDH1**	Abcam	EP1933Y	ab195254	A488	IgG
**Her-2 neu**	Abcam	24D2	ab481188	PE	IgG2a
**Vimentin**	BD Pharmingen	RV202	562337	PE	Mouse IgG1
**CK20**	Progen	IT-Ks20.10	61454	FITC	IgG1
**PD-L1**	R&D system	130021	FAB1561P	PE	Mouse IgG1
**VEGF**	R&D system	23410	IC2931G	A488	Mouse IgG2
**CD44v6**	R&D system	2F10	FAB3660A	APC	Mouse IgG1

Supplementary Table S4. CTC's DNA mutational status of all CTC lines for 6 relevant oncogenes in colorectal were analyzed by Mass-Array

		CTC-MCC-41	CTC-MCC-41.4	CTC-MCC-41.5A	CTC-MCC-41.5B	CTC-MCC-41.5C	CTC-MCC-41.5D	CTC-MCC-41.5E	CTC-MCC-41.5F	CTC-MCC-41.5G
KRAS	**Exon 2**	WT	WT	WT	WT	WT	WT	WT	WT	WT
	**Exon 3**	WT	WT	WT	WT	WT	WT	WT	WT	WT
	**Exon 4**	WT	WT	WT	WT	WT	WT	WT	WT	WT
NRAS	**Exon 2**	WT	WT	WT	WT	WT	WT	WT	WT	WT
	**Exon 3**	WT	WT	WT	WT	WT	WT	WT	WT	WT
	**Exon 4**	WT	WT	WT	WT	WT	WT	WT	WT	WT
BRAF	**Exon 15**	*c.1799T>A; p.V600E 50%*	*c.1799T>A; p.V600E 50%*	*c.1799T>A; p.V600E 50%*	*c.1799T>A; p.V600E 50%*	*c.1799T>A; p.V600E 50%*	*c.1799T>A; p.V600E 50%*	*c.1799T>A; p.V600E 50%*	*c.1799T>A; p.V600E 50%*	*c.1799T>A; p.V600E 50%*
PIK3CA	**Exon 9**	WT	WT	WT	WT	WT	WT	WT	WT	WT
	**Exon 20**	WT	WT	WT	WT	WT	WT	WT	WT	WT
PTEN	**Exon 7**	WT	WT	WT	WT	WT	WT	WT	WT	WT
AKT	**Exon 4**	WT	WT	WT	WT	WT	WT	WT	WT	WT

Supplementary Table S5. Clinical data of the 044 COLOSPOT patient at baseline.

Inclusion number		**01-044**	
**Age**		57	
**Gender**		Male	
**Cancer localization**		Transverse colon	
**TNM classification**		pTx pNx pM1	
**Metastatic sites**			
	Number	2	
	Localization	Liver	Lymph nodes
	Size	1* 22mm	Multiple* ≥15mm
**Tumor markers**			
	CA19.9	106.3 U/ml	
	ACE	3.8 ng/ml	
**Mutations**			
	KRAS	Wild type	
	BRAF	V600E mutation	

Supplementary Table S6. Normalized quantification of mRNAs expression in all colon CTC lines, HT-29, SW620 and leukocytes.

		CTC-MCC-41	CTC-MCC-41.4	CTC-MCC-41.5A	CTC-MCC-41.5B	CTC-MCC-41.5C	CTC-MCC-41.5D	CTC-MCC-41.5E	CTC-MCC-41.5F	CTC-MCC-41.5G	HT-29	SW620	Leukocytes
CTC-MCC, HT-29 & SW620	***CK19***	154.83	31.52	55.31	33.15	28.51	23.31	105.38	34.95	71.48	43.83	107.92	*0*
	EPCAM	6.72	29.84	40.61	53.38	12.93	16.71	165.62	38.80	16.78	36.45	69.73	*0*
	ECAD	0.22	0.36	0.27	0.42	0.08	0.59	0.56	1.05	1.16	0.48	1.9	*0*
	VEGF	9.74	4.46	40.30	64.13	5.14	36.31	12.66	58.19	20.53	2.08	7.64	*0*
	CMET	31.14	3.86	8.74	34.25	0.77	2.51	42.35	5.63	6.50	7.70	12.5	*0*
	SNAIL	1.41	0.45	2.11	3.88	3.84	2.33	8.04	17.41	11.00	0.05	3.82	*0*
	ALDH1	13.22	2.49	47.12	14.70	41.42	7.24	18.35	60.17	43.33	58.73	0.30	*0*
	CEACAM1	2.40	2.97	6.15	3.47	1.87	1.23	1.43	4.96	1.30	2.16	0.31	*0*
	NCAD	*0*	*0*	*0*	*0*	*0*	*0*	*0*	*0*	*0*	*0*	*0*	*0*
	CD45	*0*	*0*	*0*	*0*	*0*	*0*	*0*	*0*	*0*	*0*	*0*	4.52
CTC-MCC vs HT-29 & SW620	***IL33***	30.56	26.83	1.70	6.65	166.16	36.59	24.67	3.32	0.32	*0*	*0*	*0*
	SEMA6A	10.04	95.07	1.36	69.40	1.17	0.47	3.53	2.07	0.70	*0*	*0*	*0*
	CCND2	9.82	63.35	0.05	0.42	14.10	1.97	3.47	0.17	0.11	*0*	*0*	*0*
	OPG	1.14	3.72	10.01	11.89	25.74	1.20	0.43	39.59	36.90	*0*	*0*	*0*
	SMARCA1	*0*	*0*	*0*	*0*	*0*	*0*	*0*	*0*	*0*	6.10	5.67	*0*
	DKK1	*0*	*0*	*0*	*0*	*0*	*0*	*0*	*0*	*0*	1.73	3.79	*0*
CTC-MCC & SW620 vs HT-29	***ADAMTS6***	*0*	*0*	*0*	*0*	*0*	*0*	*0*	*0*	*0*	0.61	*0*	*0*
	GATA2	*0*	*0*	*0*	*0*	*0*	*0*	*0*	*0*	*0*	5.55	*0*	*0*
	GJB6	*0*	*0*	*0*	*0*	*0*	*0*	*0*	*0*	*0*	0.45	*0*	*0*
	TGFB2	*0*	*0*	*0*	*0*	*0*	*0*	*0*	*0*	*0*	4.21	*0*	*0*
	PTGS2	*0*	*0*	*0*	*0*	*0*	*0*	*0*	*0*	*0*	6.33	*0*	*0*
	WNT11	*0*	*0*	*0*	*0*	*0*	*0*	*0*	*0*	*0*	1.11	*0*	*0*
	BST2	*0*	*0*	*0*	*0*	*0*	*0*	*0*	*0*	*0*	2.77	*0*	*0*
	TWIST	*0*	*0*	*0*	*0*	*0*	*0*	*0*	*0*	*0*	15.93	*0*	*0*
	BCL11A	1.70	3.93	9.67	5.98	0.89	0.62	1.29	2.812	2.42	*0*	2.09	*0*
	BMP7	8.40	12.09	19.33	15.85	9.06	3.16	5.86	13.73	11.92	*0*	27.16	*0*
	FN1	0.05	142.26	1.16	31.87	2.17	0.71	5.35	3.96	1.11	*0*	2.25	*0*
	TNFRSF1B	3.00	35.51	0.11	2.21	0.87	0.64	0.14	0.02	0.024	*0*	2.57	*0*
CTC-MCC & HT-29 vs SW620	***SOX2***	*0*	*0*	*0*	*0*	*0*	*0*	*0*	*0*	*0*	*0*	1.88	*0*
	VIM	*0*	*0*	*0*	*0*	*0*	*0*	*0*	*0*	*0*	*0*	143.45	21.93
CTC-MCC differences	***DEFA6***	73.31	17.10	*0*	*0*	930.82	45.96	4.91	*0*	0.30	*0*	*0*	*0*
	ABCB1	56.22	57.14	*0*	6.48	2.86	2.89	5.13	*0*	0.02	*0*	1.59	*0*
	GAL	2.45	*0*	*0*	0	0.87	0.03	0.15	*0*	0.02	*0*	5.79	*0*

Supplementary Table S7. Proteins expression by the 9 colon CTC lines using Flow cytometry.

	CTC-MCC-41	CTC-MCC-41.4	CTC-MCC-41.5A	CTC-MCC-41.5B	CTC-MCC-41.5C	CTC-MCC-41.5D	CTC-MCC-41.5E	CTC-MCC-41.5F	CTC-MCC-41.5G
**E-cadherin**	Low	low	low	low	+	+	+	+	+
**CK20**	++	+ +	+ +	+ +	+ +	+ +	+ +	+	+
**PanCK**	++	+ +	+ +	+ +	+ +	+ +	+ +	+ +	+ +
**CD31**	-	-	-	-	-	-	-	-	-
**CD146**	-	-	-	-	-	-	-	-	-
**CD105**	-	-	-	-	-	-	-	-	-
**CD34**	-	-	-	-	-	-	-	-	-
**ALDH1**	-	-	-	-	-	-	-	-	-
**CD309**	-	-	-	-	-	-	-	-	-
**PD-L1**	-	-	-	-	-	-	-	-	-
**CXCR4**	-	-	-	-	-	-	-	-	-
**HER-2 neu**	-	-	-	-	-	-	-	-	-
**FGF-2**	-	-	-	-	-	-	-	-	-
**OPG**	-	-	-	-	-	-	-	-	-

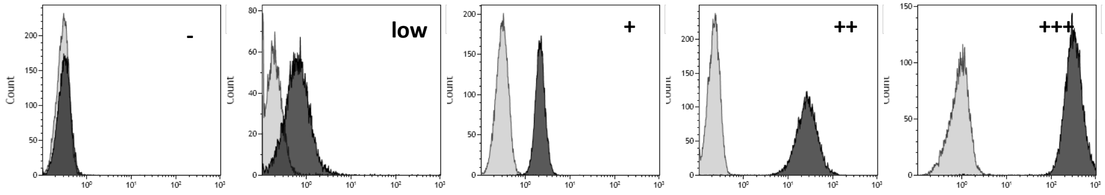

Supplement: Supplementary file 1 — Supplementary information [file 41598_2018_34365_MOESM1_ESM.pdf]
